# Specific Inhibitor of Matrix Metalloproteinase Decreases Tumor Invasiveness After Radiofrequency Ablation in Liver Tumor Animal Model

**DOI:** 10.3389/fonc.2020.561805

**Published:** 2020-11-18

**Authors:** An-Na Jiang, Jing-Tao Liu, Kun Zhao, Hao Wu, Song Wang, Kun Yan, Wei Yang

**Affiliations:** ^1^ Key Laboratory of Carcinogenesis and Translational Research (Ministry of Education/Beijing), Department of Ultrasound, Peking University Cancer Hospital & Institute, Beijing, China; ^2^ Key Laboratory of Carcinogenesis and Translational Research (Ministry of Education/Beijing), Department of Pharmacy, Peking University Cancer Hospital & Institute, Beijing, China

**Keywords:** liver tumor, radiofrequency ablation, invasiveness, specific inhibitor, matrix metalloproteinase

## Abstract

**Objective:**

To determine whether the specific inhibitor of matrix metalloproteinase (MMP)—batimastat (BB-94)—could decrease the progression of liver tumor after radiofrequency ablation (RFA) and achieve better therapeutic efficacy in an animal model.

**Methods:**

*In vitro* experiments, the proliferation of H22 liver tumor cells was detected by CCK 8 assay and cell migration was detected by Transwell method. *In vivo* experiments, H22 murine liver tumors were used. First, 32 mice with one tumor were randomized into four groups (n = 8 each group): control (PBS only), RFA alone (65°C, 5 min), BB-94 (30 mg/kg), RFA+BB-94. The growth rate of the residual tumor and the end point survival were calculated and the pathologic changes were evaluated. Secondly, a total of 48 tumors in 24 animals (paired tumors) were randomized into three groups (n = 8 each group): control, RFA alone, RFA+BB-94. Each mouse was implanted with two tumors subcutaneously, one tumor was treated by RFA and the other was evaluated for distant metastasis after applying BB-94.

**Results:**

*In vitro*, the proliferation assay demonstrated higher proliferation ability after heat treatment (0.82 ± 0.07 *vs* 1.27 ± 0.08, P = 0.008), and it could be inhibited by BB-94 (1.27 ± 0.08 *vs* 0.67 ± 0.06, P = 0.001). In the cell migration assay, the H22 cells demonstrated enhanced tumor invasiveness in the heat group than the control group (33.7 ± 2.1 *vs* 19.7 ± 4.9, P = 0.011). And it could be significantly suppressed after BB-94 incubation (33.7 ± 2.1 *vs* 23.0 ± 4.6, P = 0.009). With one tumor animal, the growth rate of the residual tumor in the BB-94+RFA group was slower than that in the RFA alone group (P = 0.003). And combination of BB-94 could significantly prolong the survival of the mice (40.3 ± 1.4d *vs* 47.1 ± 1.3d, P = 0.002). The expression of CD31 and VEGF at the coagulation margin were decreased after combined with BB-94. With two tumors animal, the growth of metastasis tumor in the BB-94+RFA group was slower than that in the RFA group (P < 0.001).

**Conclusion:**

BB-94 combined with RFA reduced the invasiveness of the liver tumor and improved the end-point survival. Our data suggested that targeting the MMP process with the specific inhibition could help to increase overall ablation efficacy.

## Introduction

Hepatocellular carcinoma (HCC) is one common malignant tumor in China and the world. Radiofrequency ablation (RFA) is a safe and effective minimally invasive therapy widely used in the unresectable hepatic tumors. However, due to the limitations of liver function, tumor size, location, and other factors, it is hard to achieve complete ablation and lead to tumor residual in some conditions. The acceleration of residual tumor progression after thermal ablation has been reported (1, 2). These data showed the residual tumor after RFA had more invasive growth, more vascular invasion and less differentiation compared with primary tumors (3). Previous studies indicated that insufficient RFA could induce over-expression of matrix metalloproteinase (MMP) (4). The expression of MMP in macrophages around liver parenchyma coagulation area increased after RFA (5).

MMPs are the member of the zinc-dependent endopeptidases family, which play an important role in the degradation of a vast number of protein targets by cleavage of internal peptide bonds (6, 7). It takes both extracellular matrix components and adhesion receptors as substrates, alters some properties of cells including the responses to the environment, and promote the migration, invasion, and metastasis of potential of tumor cells (8). MMPs could modulate the tumor microenvironment to accelerate cell growth, regulate apoptosis, regulate the bioavailability of vascular endothelial growth factor (VEGF) and promote tumor angiogenesis, and affect tumor progression (9). The specific inhibitor of MMP—Batimastat (BB-94)—is a synthetic low molecular weight metalloproteinase inhibitor, which could bound to MMPs and their catalytically active Zn atoms to inhibit the activity of MMPs (10). It has been reported in prior work that BB-94 was able to reduce tumor growth in the standard prostate cancer model (11).

Hence, we designed this study to investigate the combination of BB-94 and RFA in the treatment of hepatic tumors. We aimed to explore if BB-94 could inhibit the proliferation and migration of the residual tumors after RFA.

## Methods and Materials

### Experimental Overview

**Figure 1:**
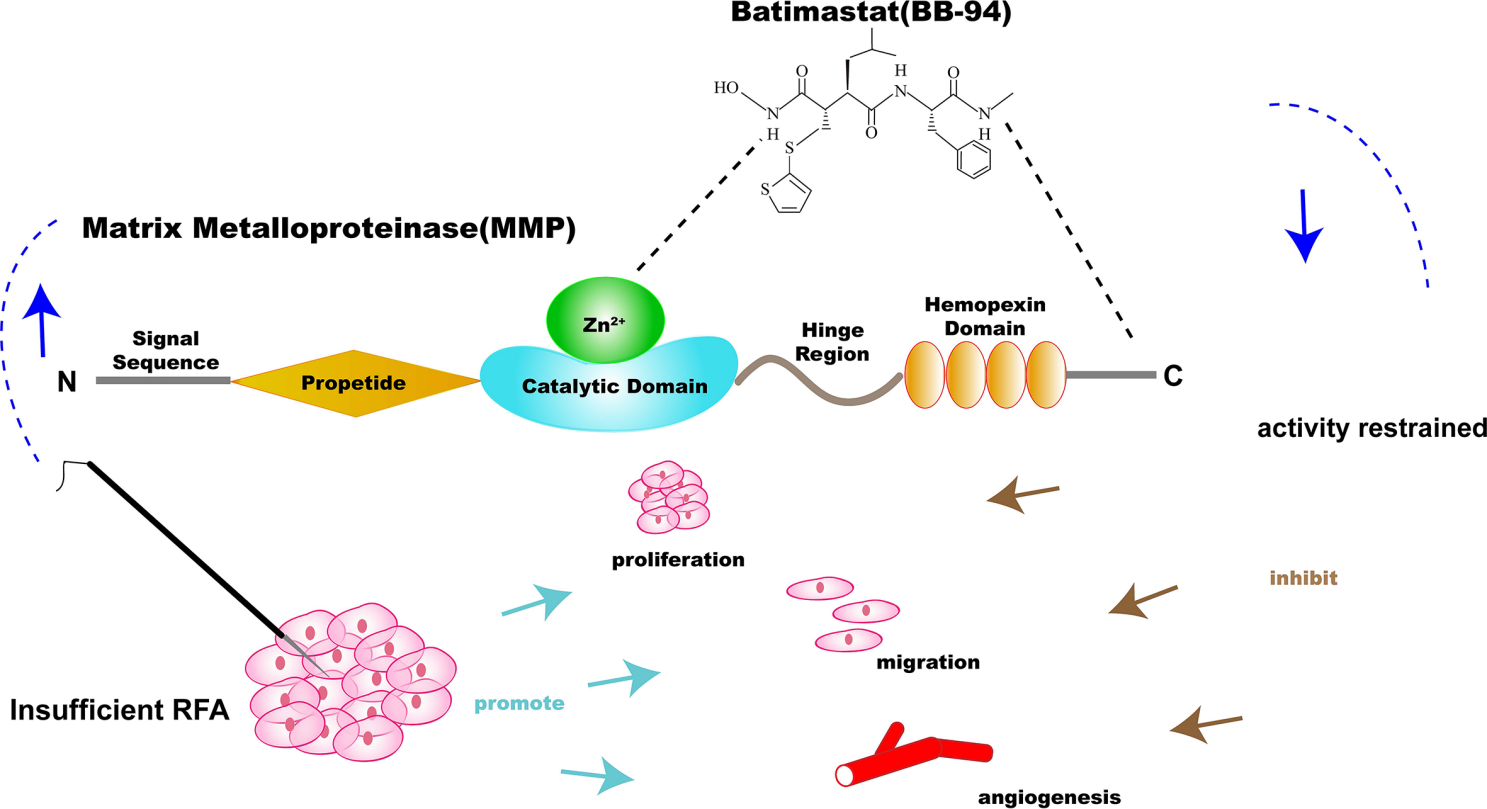
Illustration of the specific inhibitor of matrix metalloproteinase (MMP)—batimastat (BB-94)—combination therapy with RFA for solid tumor. BB-94 could inhibit the activity of MMP, which was upregulated after insufficient RFA and could promote the invasiveness of residual tumor.

The study was approved by the Institutional Animal Care and Use Committee (Peking University, Cancer Hospital) prior to the start. H22 cells (ATCC, Manassas, VA, USA) were cultured in RPMI-1640 medium containing 10% fetal bovine serum (Life Technologies, Carlsbad, CA, USA) and 0.5% penicillin-streptomycin (Life Technologies, CA, USA) at 37°C in humidified atmosphere containing 5% CO_2_. BALB/C mice (female, weighing 18–20 g, aged 6–8 weeks, Vital River Experimental Animal Technology, Beijing, China) were used in this study. The research was conducted in five phases to explore the potential synergistic effects of RFA and MMP specific inhibitors (BB-94) (APExBIO Technology, Houston, TX, USA) (**Figure 1**).

### Phase 1: Assessment of Cell Proliferation and Migration

The cell proliferation ability was detected by Cell Counting kit-8 assay (CCK-8, APExBIO Technology, Houston, TX, USA). 5 × 10^3^ H22 cells were seeded in 96-well plates of each well and incubated for 24 h. Then 10 μl CCK-8 solution was added in each well. After 4 h incubation, the absorbance value of each well was tested by microplate reader (Thermo, USA) at 450 nm (12).

The cell migration ability was assessed by Transwell assay (Corning, NY, USA). 5 × 10^5^ H22 cells were seeded in the upper chamber of each well in 16-well plates containing 8.0 μm pore size membranes with serum-free RPMI. While RPMI containing 10% fetal bovine serum was in the lower chamber of each well. After 48 h, the cells that reached the bottom of the membrane were stained with Giemsa (Sigma) and counted at ×200 magnification in five randomly selected areas per well (13).

### Phase 2: Comparison of Tumor Growth Rates

Totally, 32 mice 32 with tumors were used to compare the tumor growth. On the basis of the previous work (14), the ablation destruction was about 7 mm in diameter, while the tumor over 15 mm had a high risk of spontaneous necrosis. Accordingly, tumors at the range of 10–15 mm in diameter were selected as an appropriate size for insufficient RFA. Then the mice were randomized into the following four groups (n = 8 in each group): (a) control (PBS only); (b) RFA alone (5 min, 65°C); (c) BB-94 alone; (d) BB-94 + RFA. BB-94 (30mg/kg, 200 μl each) was injected intraperitoneally every 2 days for seven times. RFA was performed 24 h after first injection. To mimic the residual tumor during ablation of large tumors in clinical practice, about three-quarters of the tumor was completely ablated. The diameter of the residual tumor and the body weight of each mouse were measured every 2 days. The survival end point was defined as the growth of residual tumor to the diameter of 30 mm or survival of mice after treatment for 60 days, whichever was achieved first. The secondary end point was the tumor local control (i.e. no visible tumor on the abdominal wall).

### Phase 3: Assessment of Pathologic Findings

Another 12 mice from the four groups in phase 2 were sacrificed 48 h after the last injection of BB-94 (n = 3 in each group) for pathological analysis. These tumor samples were sectioned along the largest section vertical to RFA electrode. Tissue was fixed in 10% formalin overnight at 4°C, embedded in paraffin, and sliced at a thickness of 5 μm. The tissue was stained with hematoxylin-eosin for gross pathologic examination. The specific immunofluorescence (IF) staining was used to evaluate the expression of Collagen I and TGF-β. Similarly, CD31 and VEGF staining were also performed to assess the angiogenesis. Each specimen was observed for five random high-power fields per parameter and analyzed blindly to the treatment to remove the bias. The expression of CD31 and VEGF were quantified at a magnification of ×400.

### Phase 4: Comparison of Tumor Metastasis

Twenty-four mice with paired tumors (10–12 mm) were randomized into the following three experimental groups (n = 8 in each group): (a) control (no treatment); (b) RFA alone (5 min, 65°C); (c) BB-94+RFA. The mice received PBS as control. BB-94 (30mg/kg, 200 μl each) was injected intraperitoneally every 2 days for seven times. RFA was performed 24 h after first injection. Each mouse was implanted with two tumors subcutaneously on the left and right flank in this phase. For each mouse, one tumor was treated with RFA as a mimic of original site and the growth of the other site tumor, as a mimic of metastatic tumors, was then monitored afterwards. The diameter of the tumor and the body weight of each mouse were measured every 2 days. The survival end point was defined as the growth of the other site tumor to the diameter of 30 mm or survival of mice after treatment for 60 days, whichever was achieved first.

### Phase 5: Toxicity and Safety Evaluation

Twelve mice were randomized into the four groups in phase 2 but were sacrificed 48 h after the last injection of BB-94 (n = 3 in each group) to obtain important organs sample for toxicity analysis. The major organs (heart, liver, spleen, lung, and kidney) were harvested and fixed with formalin and embedded in paraffin. Then 5-μm sections were cut and stained with hematoxylin eosin (H&E) dyes for gross histopathologic analysis.

### Cell Experiments

Heat treatment: H22 cells were seeded in 6-well plates (5 × 10^4^ cells/well) for 24 h, then sealed with parafilm and submerged in a water bath set to 42°C for 6 h which was designed to mimic the effects of insufficient RFA. Meanwhile, the control temperature was set at 37°C.

BB-94 treatment: H22 cells were seeded in 6−well plates (5 × 10^4^ cells/well) for 24 h. Then the cells were treated with BB-94 (1, 2, and 4 μg/ml) and incubated at 37˚C. PBS (Life Technologies, Carlsbad, CA, USA) cultured cells were used as the control cells. After 24 h incubation, the cells were rinsed twice and replaced with fresh culture medium.

### Animal Model

For all procedures, animals were anesthetized by injecting pentobarbital sodium (45 mg/kg, chemical reagent factory of Foshan, China) intraperitoneally and sacrificed in a CO_2_ chamber; and 0.2 ml of H22 cells (at a density of 1 × 10^7^/ml) suspended in serum-free RPMI-1640 and matrigel (1:1) were injected subcutaneously into the abdominal wall with an 18-gauge needle for each tumor to establish the liver adenocarcinoma model. Animals were observed every 2 or 3 days after injection of cells to monitor the growth of the tumors and ultrasonography was performed before treatment. Thus, the solid nonnecrotic tumors were selected in the study. The longitudinal and transverse directions of the tumor was measured with mechanical calipers every 2 days in the survival studies. The measurement was performed by A-NJ and KZ, with 4 and 3 years of experience, respectively and verified by WY, with 12 years of experience, who was blinded to the treatment group. Tumor volume was calculated as (D*d)^2^*0.5, where D and d were the two diameters of the tumor measured above.

### RFA Procedure

In the animal experiments, the 17-gauge monopolar electrode (ACT1507 electrode; Valleylab, Tyco Healthcare) and the 480-kHz RFA generator (Model CC-1-220; Valleylab, Tyco Healthcare, USA) were used during RFA. The animal was shaved off on the back and applied electrolytic contact gel and then placed on the conventional metallic grounding pad (Cosman Medical, Inc. USA) to complete the RFA circuit. About 0.7 cm of the electrode tip was placed at the center of the tumor first and the RFA generator was set to the tip temperature at 65 ± 2°C and applied for 5 min.

### Statistical Analysis

In this study, SPSS 21.0 software (SPSS, Chicago, IL, USA) was used for statistical analysis. P < 0.05 was statistically significant. All continuous data were provided as means ± SD. Kruskal Wallis test was used to evaluate the significance of different treatments. When the total P was less than 0.05, Nemenyi test was used for multiple comparison. Kaplan Meier method was used for end-point survival analysis, and log-rank test was used for comparison. When P < 0.05, two specific groups were compared by the log-rank test of Bonferroni correction.

## Results

### Phase 1: Assessment of Cell Proliferation and Migration

**Figure 2:**
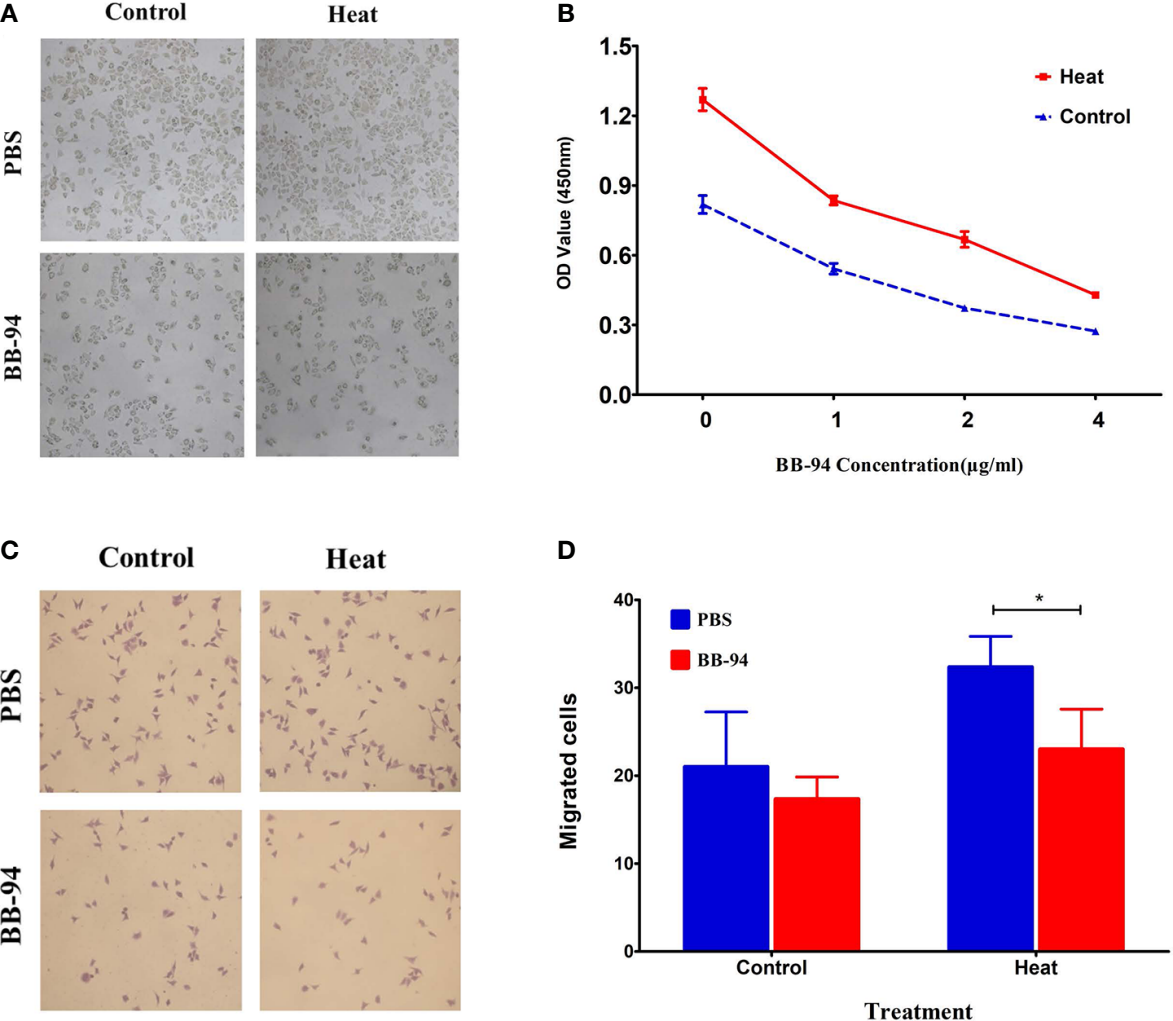
*In vitro* assessment of cell proliferation and migration after different treatment. **(A)** CCK 8 assay demonstrated the proliferation ability after heat and BB-94 treatment. **(B)** Quantitative analysis of the OD value in the different groups at different concentration of BB-94. **(C)** Transwell assay showed the migration potential of the cells in different groups. **(D)** Quantitative analysis of the migrated cells after treatment at 37 and 42°C. *P < 0.05.

*In vitro* proliferation experiment showed that the proliferation ability of H22 cells after heat treatment was significantly higher than that of the control group (1.27 ± 0.08 *vs* 0.82 ± 0.07, P = 0.008) (**Figure 2A**), and BB-94 could suppress cell growth in a dose-dependent manner (**Figure 2B**). In the cell migration experiment, the migration potential of H22 cells after heat treatment was also significantly higher than that of the control group (33.7 ± 2.1 *vs* 19.7 ± 4.9, P = 0.011) and BB-94 inhibited the migration of H22 cells (**Figure 2C**). With quantitative analysis, the heat-treated cells showed significantly lower migration potential after treated with BB-94 (33.7 ± 2.1 *vs* 23.0 ± 4.6, P = 0.009) (**Figure 2D**).

### Phase 2: Comparison of Tumor Growth Rate

**Figure 3:**
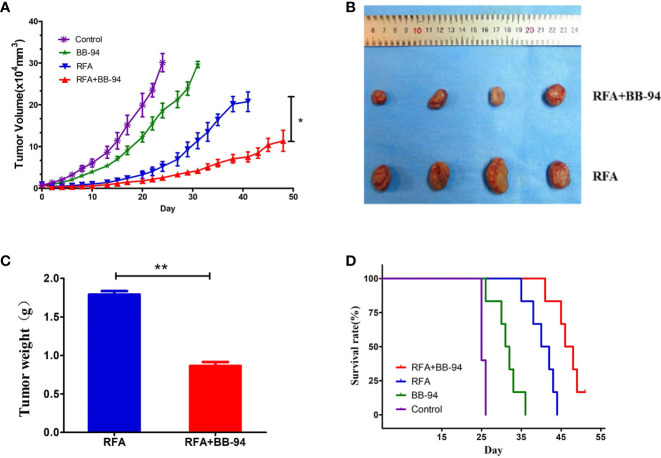
Comparison of tumor growth rate. Long-term outcomes after different treatments. **(A)** The tumor growth curves at different treatment groups. **(B)** The tumors in the RFA group and the BB-94+RFA group 30 days after RFA. **(C)** Quantitative analysis of the tumor weight in the RFA group and the BB-94+RFA group 30 days after RFA. **(D)** The survival curves at different treatment groups. *P < 0.05, **P < 0.01.

The tumor growth curves (**Figure 3A**) showed the tumor in the BB-94+RFA group grew more slowly than the RFA alone group (P = 0.003) and the tumor in the BB-94 group grew more slowly than the control group (P = 0.015). At 30 days after RFA, the volume of the residual tumor in the BB-94+RFA group was significantly smaller than that in the RFA group (**Figure 3B**) and with lighter tumor weight (1.79 ± 0.10 g *vs* 0.86 ± 0.11 g, P < 0.001) (**Figure 3C**).

Likewise, for end-point survival (**Figure 3D**), the BB-94 group (31.3 ± 1.4 days), the RFA group (40.3 ± 1.4 days), and the BB-94 + RFA group (47.1 ± 1.3 days) had better survival than the control group (25.4 ± 0.2 days) (P < 0.001). The mean survival for mice that received BB-94 was greater than that for mice without BB-94 (RFA *vs* BB-94 + RFA group, P = 0.002; BB-94 *vs* Control, P = 0.004). No organ metastasis was found at the end of follow-up.

### Phase 3: Assessment of Pathologic Findings

**Figure 4:**
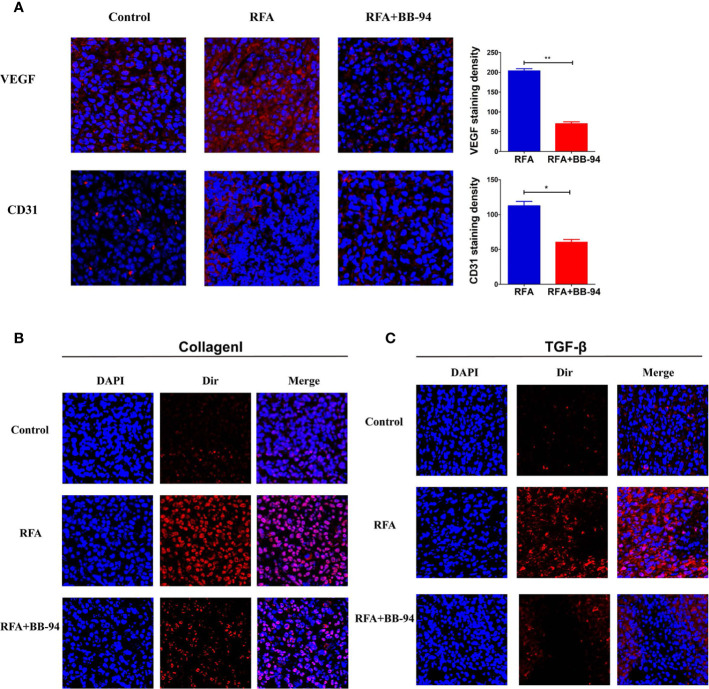
Assessment of pathologic findings was performed 48 h after the last injection of BB-94. (At the magnification of ×200) **(A)** The represented picture of VEGF and CD31 staining after different treatment and the semiqualitative analysis of VEGF and CD31 staining in the RFA group and RFA+BB-94 group. **(B)** The represented picture of Collagen I staining after different treatment. **(C)** The represented picture of TGF-β staining after different treatment. *P < 0.05, **P < 0.01.

The center of tumors treated with RFA demonstrated well-defined coagulative necrosis. IF staining in representative slides indicated a gain of more intensive VEGF and CD31 (angiogenesis) after RFA, BB-94 administration significantly inhibited the expression level of VEGF and CD31 (RFA *vs* RFA+BB-94, VEGF: 203.6 ± 12.1/high-power field *vs* 70.2 ± 10.8/high-power field, P < 0.001; CD31:112.6 ± 14.0/high-power field *vs* 60.4 ± 8.5/high-power field, P = 0.003) (**Figure 4A**). Increased collagen I deposition was observed at the periablational zone after RFA, and the expression was decreased with BB-94 administration (**Figure 4B**). Moreover, IF staining revealed that the elevated TGF-β expression in the tumor after RFA was diminished by BB-94 adjuvant treatment (**Figure 4C**).

### Phase 4: Comparison of Tumor Metastasis

**Figure 5:**
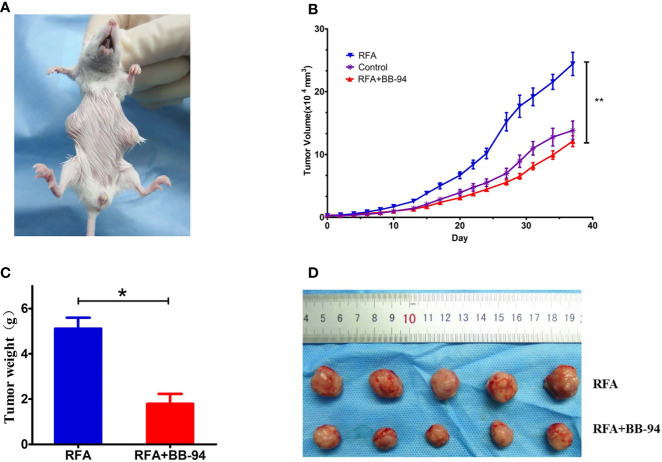
Comparison of tumor metastasis. **(A)** The paired tumors model was used in this phase. One tumor was performed with RFA and the second tumor in other site was regarded as the metastasis tumor. **(B)** The tumor growth curves of other site of tumors at different treatment groups. **(C)** Quantitative analysis of the other site of the tumor weight in the RFA group and the BB-94+RFA group 20 days after RFA. **(D)** The other site of tumors in the RFA group and the BB-94+RFA group 20 days after RFA. *P < 0.05, **P < 0.01.

The tumor growth curves indicated the growth rate of other site tumors in the RFA group was faster than that in the control group (P = 0.006) and in the BB-94+RFA group (P = 0.000) (**Figures 5A, B**). There was no significant difference between the BB-94+RFA group and the control group (P = 0.359). At 20 days after RFA, the volume of the other site tumors in the BB-94+RFA group was significantly smaller than that in the RFA group and with lighter tumor weight (1.79 ± 0.89g *vs* 5.12 ± 0.96 g, P = 0.03) (**Figures 5C, D**).

### Phase 5: Toxicity and Safety Evaluation

**Figure 6:**
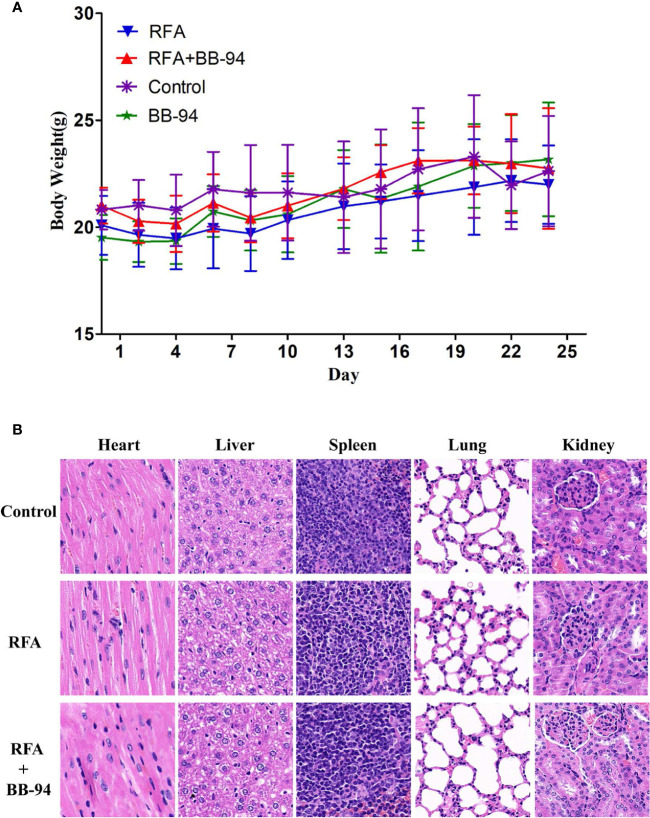
Toxicity and safety evaluation. **(A)** Changes in mice weight after treatment. During the period of follow-up, there were no obvious difference in the bodyweight change after treatment in the four experimental groups (P > 0.05). **(B)** Microscopy pathological HE staining demonstrated there was no obvious histopathological changes in heart, liver, spleen, lung, kidney in both RFA and RFA+BB-94 groups.

During the period of follow-up, there were no obvious changes in the health-related parameters after treatment including: body weight (P = 0.095, **Figure 6A**), respiratory status, eating and drinking behaviors, response to stimulations, and general activity level. Additionally, there were no obvious histopathological changes in heart, liver, spleen, lung, kidney in RFA group, RFA+BB-94 group, and control groups (**Figure 6B**).

## Discussion

Hepatocellular carcinoma (HCC) is one of the most common malignant tumors in the world with high morbidity, mortality, and increasing incidence (15). Radiofrequency ablation (RFA) is now an effective method commonly used in the local treatment of tumors. It has the advantages of minimally invasive, easy to operate, and significant curative effect. And RFA showed similar local control, long-term survival, and lower complication rates in patients with small tumors compared with hepatectomy (16). However, due to the factors such as tumor size, location, and liver function, complete ablation is often not possible. Recently, a growing number of studies have shown that residual tumors progress more rapidly after incomplete ablation (2, 17, 18).

Many studies have been reported about the underlying molecular mechanism of the increased tumor invasiveness after RFA, such as the Akt and ERK signaling pathways or through heat shock response by PKCα/Fra-1 pathway (19–21). The accelerated tumor progression after insufficient RFA was driven by many processes, but they all lead to the higher expression of MMPs. MMPs are zinc-dependent endopeptidase involved in the degradation of extracellular matrix (ECM). The MMP family is highly homologous and multidomain, which can be divided into gelatinase, collagenase, stromelysins, matrilysins, and membrane-type MMPs (22–24). MMPs are of great importance in cell proliferation, migration, differentiation, and vascularization. They could break the adhesion between cells and between cells and ECM, degrade ECM protein, promote angiogenesis, and facilitate tumor invasion and metastasis (25–28). Thus, MMPs play an important role in tumor progression after RFA and would be a potential target for treatment.

Therefore, we hypothesized that inhibiting the activities of MMPs may reduce the invasiveness of tumor after RFA. MMPs could be regulated under several levels, such as mRNA expression, proenzyme activation, and the inhibition of tissue inhibitors of metalloproteinases. For exogenous intervention, the most direct way is using specific inhibitors to inhibit the enzyme activity. MMP inhibitors have been widely studied in recent years. According to the structure, they can be divided into three categories: collagen or non-collagen peptide analogues, tetracycline derivatives, and bisphosphonates. Among the three categories, the inhibitors of collagen peptide analogues are mainly broad-spectrum inhibitors, and large sample clinical trials have been carried out. Accordingly, as one of the main MMP inhibitors, Batimastat (BB-94), was broadly studied and applied (29, 30). BB-94 is a low molecular weight peptide like collagen substrate analogue, composed of a polypeptide skeleton and an isohydroxamic acid group, which can bind to the MMP and catalytically active Zn atoms to inhibit its activity (9). Therefore, in the present study, we used BB-94 as concomitant agent in combination of RFA treatment to explore its efficacy of decreasing tumor progression.

The present study was designed based on the previous clinical and experimental findings, which indicated that insufficient RFA promoted the invasiveness of residual HCC cells *via* upregulating MMPs (4, 5). Besides, BB-94, the specific inhibitor of MMPs, was reported to inhibit tumor growth (31, 32). The present study aimed to determine the role and mechanisms of BB-94 in the process of residual tumor growth and metastasis after RFA. Initially, the inhibitory role of BB-94 in liver tumor cell growth was identified by CCK8 assays. In addition, the results demonstrated that BB-94 significantly inhibited liver tumor cell migration, as determined by Transwell assays.

We next carried out experiments *in vivo* to further corroborate experimental results *in vitro*. In the animal experiments, we demonstrated two models to evaluate the adjuvant effect of the BB-94. Based on the findings in the previous report, we were not surprised to find that combination therapy could improve the anti-tumor effect. Specifically, RFA combined with BB-94 showed slower tumor growth, while single-treatment group and control group showed positive tumor growth. Because the residual tumor after RFA was relatively small in size, the differences of early growth rate in different groups was not obvious. At 30 days after treatment, differences between RFA and BB-94 in combination with RFA began to become apparent. Best local control occurred in tumors treated with BB-94+RFA. Therefore, the end-point survival rate was consistent with the tumor growth rate, BB-94 in combination with RFA had better survival than the RFA group. No organ metastasis was found at the end of the follow up. Due to the low invasiveness of the H22 cell, we established a two-tumor model to explore the effect of BB-94 on tumor metastasis. We implanted paired tumors subcutaneously, one for ablation in original site and the other tumor was mimic the RFA stimulating distant metastasis. On the basis of findings in prior reports (33), we also found that the unablated tumor grew faster after RFA. While after applying BB-94, the tumor growth was suppressed, similarly to the untreated group (P = 0.359).

Previous studies suggested that MMP may promote tumor growth by regulating tumor angiogenesis, which was crucial for the growth and invasion of solid tumors (34–36), we then examined the two angiogenic markers—VEGF and CD31—in tumor sections. The results indicated that RFA significantly increased the expression of VEGF and CD31. MMPs may promote angiogenesis through degradation of basement membrane and ECM components, and stimulating endothelial cell migration and VEGF release, so as to promote the formation of new vessels and increase their permeability (37). And less microvessels were observed after BB-94 applied. TGF-β exhibited higher expression patterns after RFA compared to the control and relatively less staining was identified after treated BB-94. Pathologic findings also suggested that BB-94 may inhibit proliferation and migration after RFA by down regulating TGF-β signaling. RFA could destroy the tumor cells, as well as remodel the tumor microenvironment. Collagen I was one of the extrasellar proteins associated with the increased invasiveness of many solid tumors including HCC (38, 39). Collagen deposition could be seen around the ablation area, which promoted the malignant behaviors of residual tumors. And the result showed BB-94 could have pleiotropic effects, not only inhibiting MMP activity directly but also affecting collagenase production and other cellular activities. During the experiment, the BB-94 showed no special biological toxicity. We preliminarily verified the efficacy of the BB-94 through the cell and animal experiments.

There were some limitations in our study. First, we evaluated the angiogenesis and TGF-β signaling pathway under the combination of RFA and BB-94 in this study. The mechanism of other processes beside MMPs needed to be further explored in the next step. Second, we studied the effect of BB-94 in *in vivo* and *in vitro* experiments with only one cell line model. Although H22 liver tumor model in this study is a well-characterized model that commonly used in the hepatoma related tumor research, however, it should not be excluded that H22 cell is sensitive to the BB-94. So, we should apply in other models to verify the effect carefully. Furthermore, the optimal dose and time of injection is quite important in the combination therapy and need to be further explored. The detailed strategies to administration of BB-94 need to be optimized and standardized in the future. Last but not least, we established transplantation tumor model to explore the role of BB-94 in liver cancer metastasis, which might not fully recapitulate the liver cancer and the tumor microenvironment. The orthotopic model would be used to reinforce the conclusion of this study for the next experiments.

In conclusion, the specific inhibitor of MMP could help to decrease tumor invasiveness and achieve better overall outcome with combination of RFA. This adjuvant therapy might play an important role in clinical applications of RFA treatment in liver tumors.

## Data Availability Statement

The raw data supporting the conclusions of this article will be made available by the authors, without undue reservation.

## Ethics Statement

The animal study was reviewed and approved by Peking University Cancer Hospital.

## Author Contributions

A-NJ and J-TL have equal contribution to this work. All authors contributed to the article and approved the submitted version.

## Funding

This research was supported by the National Natural Science Foundation of China (81773286, 81971718) and Capital Characteristic Clinical Application Foundation (No. Z161100000516061).

## Conflict of Interest

The authors declare that the research was conducted in the absence of any commercial or financial relationships that could be construed as a potential conflict of interest.
